# A new technique to measure online bullying: online computerized adaptive testing

**DOI:** 10.1186/s12991-017-0149-z

**Published:** 2017-07-03

**Authors:** Shu-Ching Ma, Hsiu-Hung Wang, Tsair-Wei Chien

**Affiliations:** 1 College of Nursing, Kaohsiung Medical University, Kaohsiung, Taiwan; 2 Nursing Department, Chi-Mei Medical Center, Tainan, Taiwan; 3 Research Department, Chi-Mei Medical Center, 901 Chung Hwa Road, Yung Kung Dist., Tainan, 710 Taiwan; 4 Department of Hospital and Health Care Administration, Chia-Nan University of Pharmacy and Science, Tainan, Taiwan

**Keywords:** Computerized adaptive testing, Non-adaptive testing, Item response theory, The Negative Acts Questionnaire-Revised, workplace bullying

## Abstract

**Background:**

Workplace bullying has been measured in many studies to investigate mental health issues. None uses online computerized adaptive testing (CAT) with cutting points to report bully prevalence at workplace.

**Objective:**

To develop an online CAT to examine person being bullied and verify whether item response theory-based CAT can be applied online for nurses to measure exposure to workplace bullying.

**Methods:**

A total of 963 nurses were recruited and responded to the 22-item Negative Acts Questionnaire-Revised (NAQ-R). All non-adaptive testing (NAT) items were calibrated with the Rasch rating scale model. Three scenarios (i.e., NAT, CAT, and the randomly selected method to NAT) were manipulated to compare their response efficiency and precision by comparing (i) item length for answering questions, person measure, (ii) correlation coefficients, (iii) paired *t* tests, and (iv) estimated standard errors (SE) between CAT and the random to its counterpart of NAT.

**Results:**

The NAQ-R is a unidimensional construct that can be applied for nurses to measure exposure to workplace bullying on CAT. CAT required fewer items (=8.9) than NAT (=22, an efficient gain of 60% =1–8.9/22). Nursing measures derived from both tests (CAT and the random to NAT) were highly correlated (*r* = 0.93 and 0.96) and their measurement precisions were not statistically different (the percentage of significant count number less than 5%) as expected, but CAT earns smaller person measure SE than the random scenario. The prevalence rate for nurses was 1.5% (=15/963) when cutting points set at −0.7 and 0.7 logits.

**Conclusion:**

The CAT-based NAQ-R reduces respondents’ burden without compromising measurement precision and increases endorsement efficiency. The online CAT is recommended for assessing nurses using the criteria at −0.7 and 0.7 (or <30 and <60 in summed score) to identify bully grade as one of the three levels (high, moderate, and low). The bullied nurse can get help from a psychiatrist or a mental health expert at an earlier stage.

**Electronic supplementary material:**

The online version of this article (doi:10.1186/s12991-017-0149-z) contains supplementary material, which is available to authorized users.

## Background

During the last 20 years, the prevalence rate of workplace bullying has been reported in a range of different studies to investigate mental health issues [1–3]. Despite all this attention on the bully phenomenon, the criteria of cutting points indeed influence the calculation of prevalence rate on workplace bullying.

The prevalence rate of bullying, using the same bully scale of the 22-item Negative Acts Questionnaire-Revised (NAQ-R) with examinee’s self-labeling (i.e., with a single quest to answer whether she/he is a bullied victim [4, 5]), was, respectively, reported at 24% for hospital nurses [2], higher than seen in studies of Japanese nurses (19%) [3], and Italian employees (15.2%) [4], and workers in general services (2–17%) [1]. Nielsen et al. [6] addressed that self-labeling with definition studies yielded far lower estimates of bullying than self-labeling studies without definitions. The findings for the prevalence rate on workplace bullying would be thus biased and overestimated without definitions when self-labeling bullied perception.

### Common cutting points are required

For studies using the behavioral method (i.e., with several items to respond with regard to encountered negative acts or behaviors in a workplace [1, 7], like the NAQ-R) with an operational criterion, prevalence rates seem to vary between 3 and 17%, depending on the cutoff criterion utilized [8]. Unfortunately, no such a common cutting point for calculating the bully prevalence rate was applied to the NAQ-R till now. A comparison between derived score levels and the suggested best cutoff points can help clinicians evaluate examinees at risk of an incidence [9, 10], and multiple cutoff points are usually more powerful and useful than one single cutoff point [11, 12]. How to determine appropriate cutting points for the NAQ-R is an issue of the current study.

### Cutting points are required for computerized adaptive testing

The NAQ-R is evident of a unidimensional construct and can be applied to measure exposure to workplace bullying through the computerized adaptive testing (CAT) administration [2]. The CAT requires fewer items to answer than the traditional pen-and-paper approach (an efficiency gain of 32%), suggesting a reduced burden for respondents [2]. However, the CAT-based NAQ-R is just administered on a computerized nursing cart (i.e., not an online CAT version) and is not set with multiple cutting points to help clinicians evaluate examinees at risk of an incidence, especially because each person answers a different number of items on the CAT. Determining cutting points is thus a critical issue for the NAQ-R CAT.

### Computerized adaptive testing

Computerized adaptive testing (CAT) is based on item response theory (IRT)_test that adapts to the examinee’s ability level. The computer follows an IRT-based algorithm that offers the patient the next not-too-hard-and-not-too-easy item. So, only the fewest possible items are offered per patient, resulting in less respondent burden and even more accurate outcomes [2]. As with all forms of Web-based technology development, there is no *online* CAT assessment applied to the NAQ-R till now.

### Objectives

First, we verify whether the NAQ-R is a unidimensional construct. Second, we determine a set of cutting points that can be used for computing a prevalence rate at workplace on CAT. Third, we compare CAT with non-adaptive testing (NAT) and the randomly selected method to NAT on efficiency and precision. Fourth, we developed an online CAT for nurses to measure exposure to workplace bullying.

## Methods

### Study participants

The study sample was recruited from three hospitals (Hospital A: 1236-bed medical center; B: 265-bed local hospital; C: 877-bed region hospital) in southern Taiwan in the summer of 2012. No incentive for participation was offered. A total of 970 copies of the bully questionnaire were validated with a return rate of 96.3%.

This study was approved and monitored by the Research Ethics Review Board of the Chi-Mei Medical Center. Demographic data were anonymously collected: gender, work tenure in hospitals of all types, age, marital status, and education level.

### Scales used for reporting exposure to bullying

The 22-item NAQ-R with 5 response alternatives (1 = never, 2 = occasionally, 3 = monthly, 4 = weekly, 5 = daily) was used to measure exposure to workplace bullying within the past 6 months. With permission from the author [13], the NAQ-R was professionally translated into Chinese by authors in Taiwan using a back-translation technique (English–Chinese–English).

### Dimensionality

Tennant and Pallant [14] suggested three steps that should be applied to assess scale unidimensionality: (1) conduct prior testing using Horn’s parallel analysis [15] for ensuring that unidimensionality is retained, (2) use Rasch [16] fit statistics ranging from 0.5 to 1.5 [17, 18] to determine the usefulness of the one-dimensional scaling, and (3) run post hoc tests using Rasch standardized residual loading [19] (i.e., |*Z*| < 2.0) across items to inspect the convergent validity, and Smith [20] independent *t* tests to compare estimates of the percentages (<5%, within ±1.96) and verify invariance of Rasch model. A dimension coefficient (>0.67, DC) suggested by Chien [21] was used for identifying a single-dimensional scale. Point-biserial correlation coefficients on items (PTME, the Pearson correlation between the observations of an item and the item difficulties that is like factor loading in exploration factor analysis) >0.40 was reported to support scale dimensionality.

### Cutting points used for the NAQ-R

According to the literature [22–24], as a scale’s reliability (i.e., Cronbach’s α) increases, so does the person-number of ranges that can be confidently distinguished. Measures with reliabilities of 0.67 will tend to vary within two groups that can be separated with 95% confidence; 0.80 will vary within three groups; 0.90, within four groups; 0.94, within five groups; 0.96, within six groups; 0.97, within seven groups; etc. [25].

More conservative to compute the number of the strata, the scale reliability was referred to the Rasch person separation reliability, and then referred to the Rasch threshold difficulty guideline [26] with an appropriate distance between two thresholds ranging from 1.4 to 5.0 logits.

An equal sample size in each stratum suggested by Maslach et al. [27] was applied to determine cutting points. Accordingly, a threshold at zero logits is suggested for two strata, −0.7 and 0.7 (=1.4 − logit difference with probabilities at 0.33 and 0.67 = 1 − exp (−0.7)/[1 + exp (−0.7)] for three strata, −1.1, 0.0, and 1.1 (=1.1 − logit difference with probabilities at 0.25, 0.50, and 0.75 = 1 − exp (−1.1)/[1 + exp (−1.1)] for four strata, and −1.4, −0.4, 0.4 and 1.4 (=1.0 − logit difference with probabilities at 0.20, 0.40, 0.60 and 0.80 = 1 − (−1.4)/[1 + exp (−1.4)] for five strata.

### Comparison of efficiency and precision using CAT algorithm

**Fig. 1:**
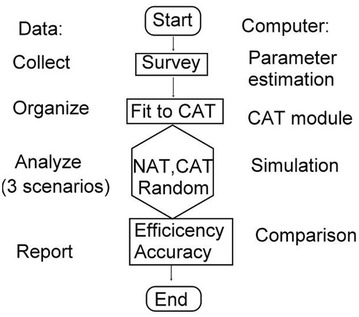
Flowchart in comparison with CAT efficiency and accuracy

Three scenarios (i.e., NAT, CAT, and the randomly selected method to NAT) were manipulated to compare their response efficiency and precision by comparing (i) item length for answering questions, person measure, (ii) correlation coefficients and (iii) Smith’s paired *t* tests [20], and (iv) estimated standard errors (SE) between CAT and the random to its counterpart of NAT (Fig. 1).

We ran an author-programed VBA (Visual Basic for Applications) module in Microsoft Excel. Rasch person separation reliability yielded from the NAQ-R of the study by Winsteps (i.e., excluding all extreme scores summed to zero) was used to determine the CAT termination criterion using the standard error of measurement (SEM = SD * √1 − reliability). Another termination criterion is the mean of the last five change differences between the pre- and post-estimated abilities on each CAT <0.05.

The minimum number of questions required for completion was set at 7 (7/22 items on NAQ-R item length = 30%). The first item was randomly selected from the 22 items when starting the CAT. The provisional measures were estimated by the maximum log-likelihood estimation (MLE). The next question selected was the one with the most information obtained from the remaining unanswered items, interacting with the previously provisional person measures.

### An online CAT was designed for smart phones

An online CAT was designed for examinees to report their bully scores in a unit of logit (log odds). The 22 items with their threshold difficulties (calibrated by Rasch Winsteps) and their responsive audios and pictures were uploaded to the website. The rules of the first and the next selected CAT item and the termination criteria are like the aforementioned simulation method.

### Statistical tools and data analyses

SPSS 15.0 for Windows (SPSS Inc., Chicago, IL) and MedCalc 9.5.0.0 for Windows (MedCalc Software, Mariakerke, Belgium) were used to calculate (1) Cronbach’s α, (2) dimension coefficients, and (3) correlation coefficients between estimated person measures for CAT and the random to its counterpart of NAT. Independent *t* tests were used to compare (4) the ratios of the different paired person measures. Rasch Winsteps was used for producing (5) person separation reliability. The prevalence rate of workplace bully is calculated by the formula (=the number of bullied grade excluded from the low stratum divided by the sample).

## Results

The sample of 963 nurses was obtained from the study. The mean age of the participants was 32.7 (±5.8) years, 96% (*n* = 924) were female, and >57.5% (*n* = 554) were unmarried (Table 1).

**Table 1:** Demographic characteristics of the participants (*n* = 963)

Variable	Category	Number	%
Hospital			
	Hospital A	543	56.4
	Hospital B	324	33.6
	Hospital C	96	10.0
Gender			
	Male	39	4.0
	Female	924	96.0
Education			
	High school	6	0.6
	College	465	48.3
	University	475	49.3
	Graduate school	17	1.8
Marriage			
	Unmarried	554	57.5
	Married	405	42.1
	Divorced	4	0.4
Nursing grade			
	N0	34	3.5
	N1	282	29.3
	N2	317	32.9
	N3	244	25.3
	N4	86	8.9
Title			
	Nurse	773	80.3
	Chief	170	17.7
	Leader	8	0.8
	Others	12	1.2

Age and tenure	Mean	SD	Range
Age (years)	32.7	5.8	23–55
Out of hospital (months)	21.9	34.7	0–240
Within hospital (months)	89.6	49.7	3–378

### Dimensionality

The NAQ-R can be unidimensional because

1. one factor was extracted using parallel analysis;
2. all Infit and Outfit mean squares for the 22 items are in a range of 0.5–1.5 (in the Infit column in Table 2; Fig. 2);

**Table 2:** One factor extracted from the Negative Acts Questionnaire-Revised (NAQ-R) scale with mean square between 0.50 and 1.50

During the last 6 months, how often have you been subjected to the following negative acts in the work place?						
Item	Feature	Difficulty			MNSQ	loading
	Type^a^	Delta	SE	PTME	Infit	Z
21. Being exposed to an unmanageable workload	1	1.5	0.14	0.53	0.97	1.57
22. Threats of violence or physical abuse or actual abuse	3	1.5	0.14	0.51	0.95	0.26
9. Intimidating behaviors such as finger-pointing, invasion of personal space shoving, blocking your way	3	1.29	0.13	0.51	1.07	−0.41
8. Being shouted at or being the target of spontaneous anger	3	0.87	0.12	0.56	1	−0.26
19. Pressure not to claim something to which by right you are entitled	1	0.74	0.11	0.62	0.76	0.81
3. Being ordered to do work below your level of competence	1	0.5	0.11	0.59	1.04	−0.41
18. Excessive monitoring of your work	1	0.23	0.1	0.61	1.14	1.08
5. Spreading of gossip and rumors about you	2	0.16	0.1	0.61	1.09	−0.66
15. Practical jokes carried out by people you don’t get along with	2	0.06	0.1	0.67	0.83	1.54
16. Being given tasks with unreasonable deadlines	1	−0.06	0.09	0.66	0.98	1.05
14. Having your opinions ignored	1	−0.08	0.09	0.68	0.87	0.78
20. Being the subject of excessive teasing and sarcasm	2	−0.11	0.09	0.67	0.93	1.17
2. Being humiliated or ridiculed in connection with your work	2	−0.17	0.09	0.62	1.21	−0.60
17. Having allegations made against you	2	−0.19	0.09	0.66	1.02	0.96
6. Being ignored or excluded	2	−0.24	0.09	0.68	0.92	−1.21
12. Being ignored or facing a hostile reaction when you approach	2	−0.39	0.09	0.7	0.94	−0.93
11. Repeated reminders of your errors or mistakes	2	−0.41	0.09	0.68	1.07	−1.24
10. Hints or signals from others that you should quit your job	2	−0.83	0.08	0.69	1.1	−0.84
1. Someone withholding information which affects your performance	1	−0.89	0.08	0.69	1.16	−1.08
13. Persistent criticism of your errors or mistakes	2	−0.9	0.08	0.74	0.83	0.75
4. Having key areas of responsibility removed or replaced with more trivial or unpleasant tasks	2	−1.29	0.08	0.71	1.11	−1.24
7. Having insulting or offensive remarks made about your person, attitudes, or your private life	2	−1.29	0.08	0.7	1.3	−1.11
Minimum		−1.29	0.08	0.51	0.76	−1.24
Maximum		1.50	0.14	0.74	1.30	1.57

Threshold difficulties are −3.26, −0.71, 0.71, 3.25

^a^Type: *1* work-related bullying; *2* Person-related bullying; *3* physically intimidating bullying

**Fig. 2:**
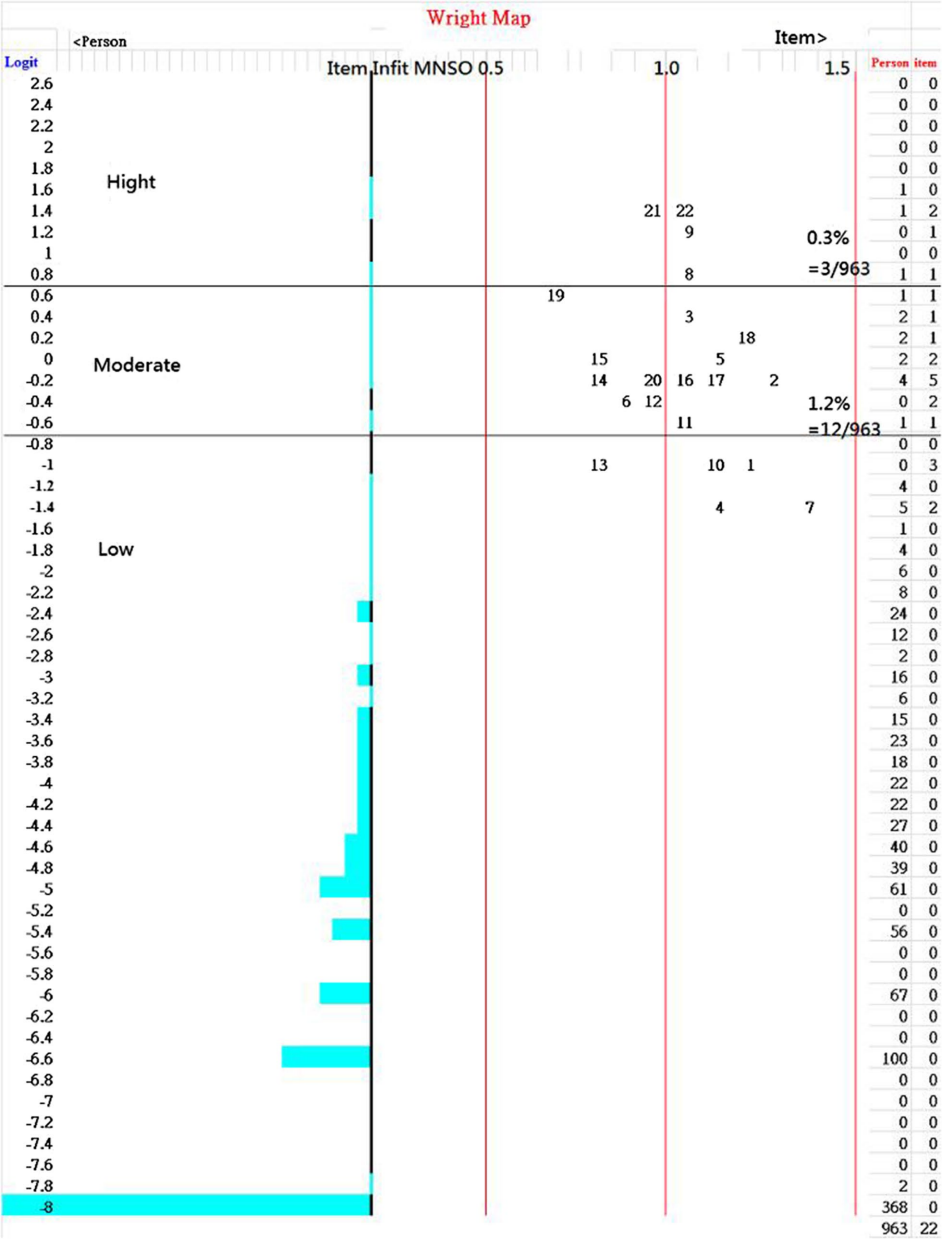
Item and person dispersion on an interval logit continuum scale
3. item loadings from the Rasch PCA of residuals on the first contrast are standardized (i.e., (loading − mean)/SD) within −1.24 and 1.57 (within ±2.0 in the Z column in Table 2); PTME are between 0.51 and 0.74 (in the PTME column in Table 2).

Rasch person separation reliability = 0.84, Cronbach α = 0.96, DC = 0.88 (>0.67), and Smith’s *t* test of proportions [20] is near to zero (=1.14% = 11/963) outside the range ±1.96. In addition, category structure for the NAQ-R displays the monotonically increasing threshold (−3.26, −0.71, 0.71, 3.25 logits) in compliance with Linacre’s guidelines [26] at least distance ranging from 1.4 to 5.0 logits.

### Cutting point determination

The person separation reliability for the NAQ-R is 0.84, indicating that three strata can be separated with thresholds at −0.7 and 0.7. Prevalence rate of workplace bully is 1.5% (=0.3% + 1.2%), see Fig. 2.

### Comparison of efficiency and precision

**Fig. 3:**
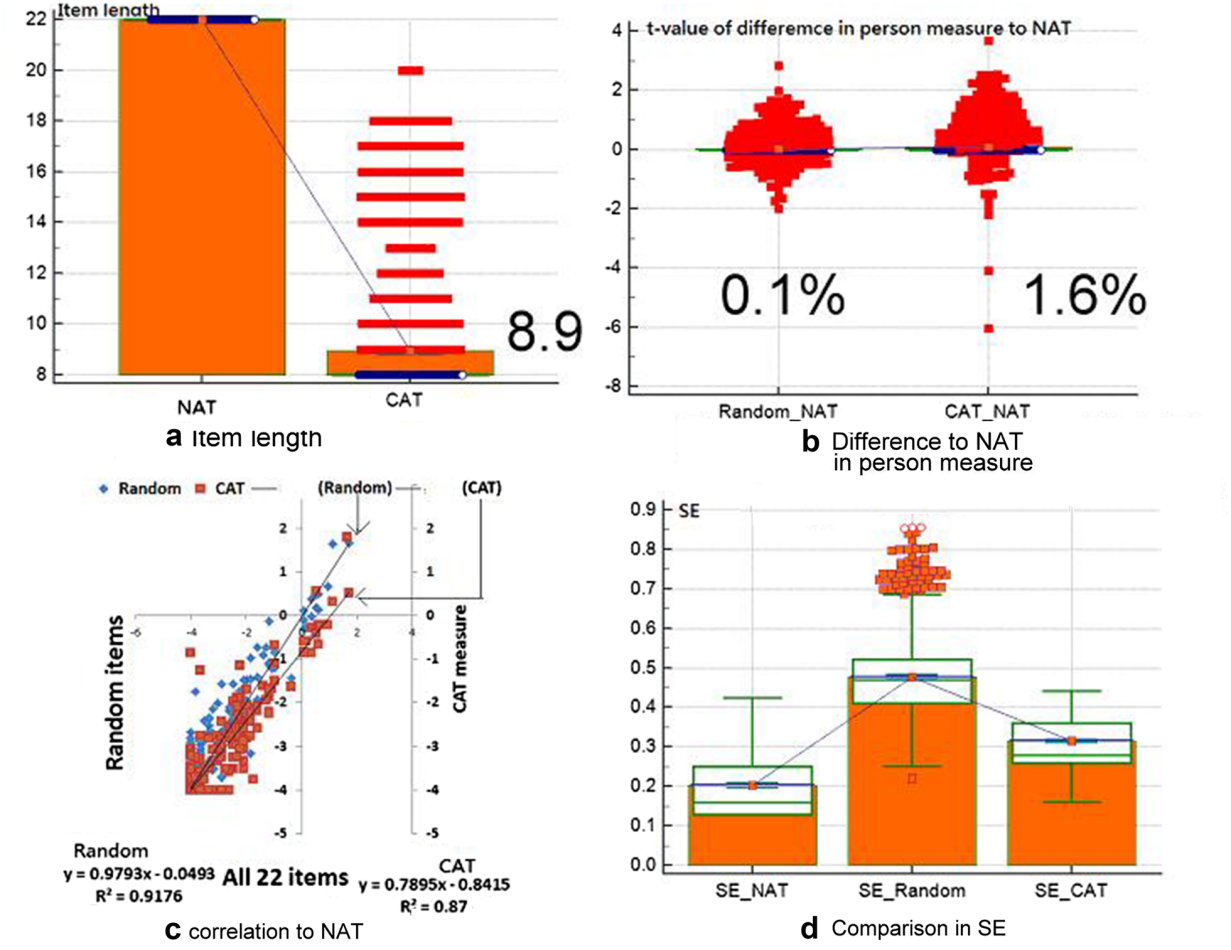
Comparison in efficiency and accuracy among scenarios

The CAT required substantially fewer items (mean = 8.9; SD = 2.4; SE = 0.08; 95% CI 8.78–9.09) than did NAT (=22) and provided an efficient gain in test length of 0.60 (=1–8.9/22), see Fig. 3 in panel a. Person measures from CAT did not statistically differ from NAT because (1) Smith’s *t* test of proportions [20] is 1.6% (=15/963 < 5%), see Fig. 3 in panel b, and (2) correlation coefficient = 0.93 (=√ÔR-square = √0.87, see Fig. 3 in panel c). As compared to the random scenario, CAT earns a set of smaller SE, see Fig. 3 in panel d.

### Online NAQ-R assessment

**Fig. 4:**
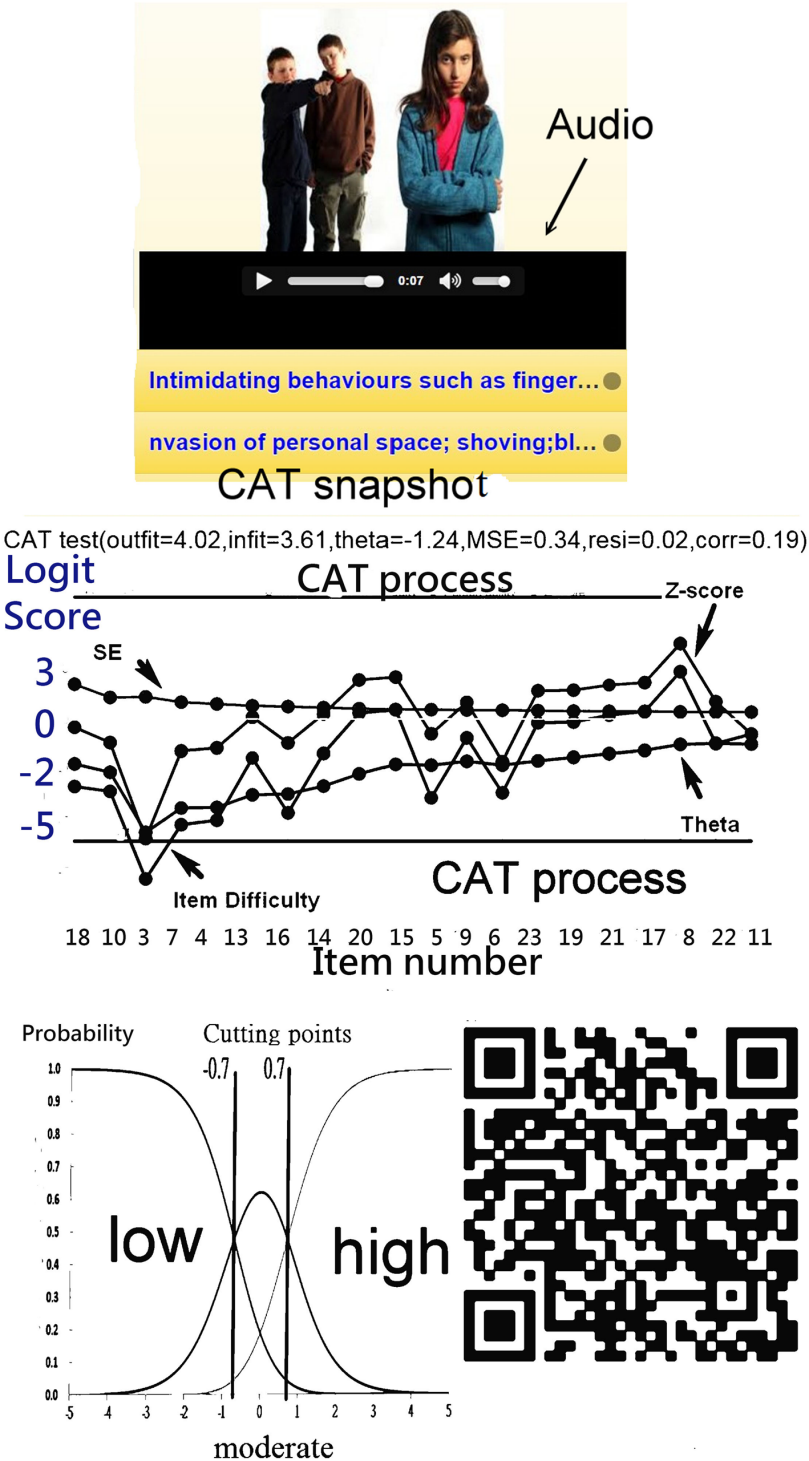
A snapshot of online CAT-based NAQ-R assessment

By scanning a QR-code (Fig. 4 at right bottom), the NAQ-R item appears on the smartphone. We developed an online CAT module to demonstrate the assessment in action. The CAT processed each nurse item-by-item with picture animations (Fig. 4 at top). Adaptive item selection is based on maximizing information across unanswered items. The measurement of standard error (MSE) for each subscale decreased when the number of the items increased (Fig. 4). The result with a person measure and the bully grade (i.e., low, moderate, or high) instantly shows on smartphone (Fig. 4).

## Discussion

### Key findings

The results from this study indicate that the 22-item NAQ-R is unidimensional. A set of cutting point at −0.7 and 0.7 logits were determined for future use in workplace bullying surveys. The prevalence of bullying for the study sample was 1.5%. The CAT is 60% more efficient for answering questions and achieved similar precision in measurements as did NAT. An available-for-download online CAT NAQ-R APP for nurses was suited for smartphones (Additional file 1).

### What this adds to what was known

Consistent with the literature [2, 28–32], the 22-item NAQ-R can be unidimensional. The efficiency of CAT over NAT was supported. We confirm that CAT-based NAQ-R requires significantly fewer answered items to measure explosion of workplace bully than NAT without compromising its measurement precision.

### What it implies and what should be changed?

#### Cutoff point recommended for calculating bully prevalence rate

According a study in Belgian employees [33], six different groups of respondents were identified based on their exposure to negative behaviors: (1) not bullied (35%), (2) limited work criticism (28%), (3) limited negative encounter (17%), (4) sometimes bullied (9%), (5) work-related bullying (8%), and (6) victims of bullying (3%). Too many grades is hard to help clinicians evaluate examinees at risk of an incidence [9, 10]. A single cut point of >–4.2 logits (or >30 in summation) for the NAQ-R was proposed [2]. However, multiple cutoff points are usually more powerful and useful than one single cutoff point [11, 12]. Maslach et al. [27] suggested setting an equal sample size in each stratum as a way to determine cutting points.

At the end of 2016, more than 10,977 papers were found in a search with keyword “cut point.” None discussed the determination of cutting points used for CAT with different item lengths for a respondent. Frequently, we usually do not know the patient’s true- and false-positive disease-specific status, like the NAQ-R. The issue we face in clinical settings is how to identify the degree of patient incident problems. Through this study, if cutting points at −0.7 and 0.7 logits are selected for the NAQ-R, the raw score in cutting points can be transformed by the formula (=total score × the probability at 0.33 and 0.67), whereas 0.33 comes from the equation exp (−0.7)/(1 + exp (−0.7)) and 0.67 is from the equation 1 − exp (−0.7)/(1 + exp (−0.7)), total score = 88 when 5-point (from 0 to 4) 22-item NAQ-R is defined beforehand. The cutting points in raw score can be set at <30 (=88 × 0.33), and ≥60 (=33 × 0.67) to separate three strata in bully degree. The prevalence rate is easy to calculated and compared either with paper-and-pen format or with CAT in future.

#### Online CAT assessment

At the end of 2016, 757 papers were collected in US National Library of Medicine National Institutes of Health (pubmed.org) when searching keywords: computer adaptive testing. None was applicable using an online assessment suited for smartphones until the online skin cancer CAT was published [32]. We do ensure that more papers in future will be published on the usefulness of online CAT as with all forms of Web-based technology are rapidly increasing [34].

#### Unidimensional scale detection

Many studies [21, 35–38] reported the issue of scale unidimensionality detection. From the Library of PubMed and BioMed Central, we got 1005 and 333 papers with the keyword “unidimensionality,” 4688 and 745 results for “bully.” In the current study, we demonstrated the method Tennant and Pallant [14] suggested using three steps to assess scale unidimensionality: (1) conduct prior testing using Horn’s parallel analysis, (2) use Rasch fit statistics, and (3) run post hoc tests using Rasch standardized residual loading, and Smith [20] independent t tests to compare estimates of the percentages (<5%, within ±1.96). In addition, the dimension coefficient (≥0.67, DC) and PTME (>0.40) included in detecting scale unidimensionality are recommended to readers.

### Strengths of this study

Four goals have been reached in this study: (1) we verified the 22-item NAQ-R is unidimensional, (2) cutting points at −0.7 and 0.7 logits were recommended to future studies in computing bully prevalence rate at workplace, (3) CAT gains 60% efficient than did NAT, and (4) online CAT is applicable in practice. Among them, the reason for 60% efficient than did NAT is because we added another termination rule in CAT: the mean of the last five change differences between the pre- and post-estimated abilities on each CAT less than 0.05. The termination rule of detecting the last five change differences in estimated abilities less than 0.05 makes the item length less than that in other studies [2, 28–31]. It is because many low grade of workplace bully were found and led to short item length required to complete the CAT. Around 82.6% (=795/964) terminated CAT at eight items. A total of 368 nurses responded to all items with zero (i.e., never). If all CAT cases are controlled by the only termination rule of SE less than 0.44 (=SQRT (1 − 0.8) = SQRT (1 − reliability)), the precision measured by SE on CAT (in panel D in Fig. 3) will be substantially higher than the dual stop conditions we did in this study.

In addition, the online CAT with audio and picture animations is available for interested readers to practice if scanned on the QR-code in Fig. 3, which is rare in any previously published articles.

Furthermore, cutting points set at −0.7 and 0.7 logits with an equal stratum member size might be generalized to other incidences or diseases when the patient’s true- and false-positive disease-specific status is not known beforehand. Like the NAQ-R, we merely intend to identify the grade of the incidence and compare to the norm.

### Limitations of the study

Several issues should be considered more thoroughly in further studies. First, many female nurses (96%) in sample let us not identify differential item functioning (DIF) on gender. Second, the low bully prevalence rate (1.5%) was reported here as compared to the previous papers at 24% for hospital nurses [2], higher than seen in studies of Greek nurses (30.2%) [39], Japanese nurses (19%) [3], Korean nurses (17.2%) [40], and Italian employees (15.2%) [4], and workers in general services (2–17%) [1]. One ensured reason is attributable to different cutting points and self-labeling definitions. For instance, one [40] defined a victim of workplace bullying if subjects had experienced at least 2 of the 22 negative acts from NAQ-R by a colleague every day or every week in the past 6 months. Another [39] used an additional question “Have you been bullied at work?”. Valid criteria are thus urgently required to classify levels of incidence and to calculate the prevalence rate of workplace bully. Accordingly, the study cannot be generalized to others.

More studies are needed to assess the generalizability of the study with different samples using the same cutting points and the same version of NAQ-R. Third, the online CAT is not equipped with much functionality as we expected in practice, such as protecting cheating behaviors and detecting aberrant responses that are required to be in future advanced versions. Fourth, although the scale’s Cronbach’s α coefficients was 0.96, we conservatively determined that the scales’ person strata were three according to Rasch separation reliability = 0.84 and the literature [22–25]. Multiple cutoff points are not limited to three strata if the separation index reaches an extremely higher level, which will affect the determination of appropriate cutting points for the NAQ-R.

## Conclusions

The CAT-based NAQ-R forming a unidimensional construct reduces respondents’ burden without compromising measurement precision and increases endorsement efficiency. The online NAQ-R module developed by the authors is recommended for assessing nurses or other workers using the criteria at −0.7 and 0.7 (or <30 and <60 in summed score) to identify bully grade as one of the three levels (high, moderate, and low). The bullied nurse can get help from a psychiatrist or a mental health expert at an earlier stage.

## Additional file

**Additional file 1.** Online bully CAT video at https://youtu.be/te9Gmpi9q8w.

## Abbreviations

APP: application
CAT: computer adaptive testing
CTT: classic test theory
DC: dimension coefficients
DIF: differential item functioning
IRT: item response theory
MLE: maximum likelihood estimation
MNSQ: mean square
MSE: mean-squared error
NAT: non-adaptive testing
NAQ-R: Negative Acts Questionnaire-Revised
PCA: principal component analysis
PTME: point-biserial correlation coefficients on measures
SD: standard deviation
SE: standard error
SEM: standard error measurement
VBA: visual basic for applications
